# Strategies for recruiting diverse young adults into aging research

**DOI:** 10.1002/alz.087701

**Published:** 2025-01-09

**Authors:** Haydee Izurieta Munoz, Antonio Casas, Myles Gaddy, Selena Hernandez Sanchez, Leigh A. Johnson

**Affiliations:** ^1^ University of North Texas Health Science Center, Fort Worth, TX USA

## Abstract

**Background:**

In aging research, there is an increasing focus on the decades leading up to changes in cognition. Biomarker studies have suggested that biochemical brain fluctuations can occur up to 20 years prior to clinical symptoms. Including populations of adults who are 20 years away from any cognitive symptomatology in aging research can shed a light on the longitudinal factors involved in brain aging. The aim of this study was to describe and evaluate the methods of recruiting ethnically diverse younger adults (30‐49 years old) into aging research.

**Method:**

The Health & Aging Brain Study‐ Health Disparities (HABS‐HD) study is a community based longitudinal study of cognitive aging in a multiethnic cohort. Participants must undergo an interview, neuropsychological testing, a blood draw, an MRI scan and PET scan of the brain. The study began in 2017, with the goal of enrolling 3000 diverse participants 50 years or older. Thanks to a grant from the National Institute of Aging, the study began enrolling 1500 participants 30‐49 years old in September 2022. The previous outreach strategies focused on older adults had to be modified and updated to cater to the younger group. The outreach team developed, implemented and tracked a variety of strategies from people signing up from the study through enrollment.

**Result:**

From September 2022 through November 2023, 541 people 30‐49 signed up to be part of the study. 69.7% were female and 30.3% were male, with a mean age of 40 (SD = 5.9). Of those 541 sign ups, 461 participants scheduled an appointment and 332 came in for their study visit (61.4% enrollment rate). The sample included 207 Black/African Americans, 234 Hispanics, 93 Non‐Hispanic Whites and 7 participants of another race. Enrollment data showed that the younger group were recruited mostly through personal referrals (40.1%) and outreach events (38.1%).

**Conclusion:**

The study team had to modify approaches to recruitment in order to enroll a younger cohort into the HABS‐HD study, since approaches used previously for older adult were not sufficient. Personal referrals were the biggest drivers of successful enrollment, which suggests that personal trust is crucial in recruiting a novel cohort.

